# Supplementary material for: Gateway analysis reveals transient molecular programs at cell-fate transitions
Source: bioRxiv. 2026 Mar 16:2026.03.12.711328. Preprint. [Version 1] doi: 10.64898/2026.03.12.711328 (PMC13015526; doi:10.64898/2026.03.12.711328)
Supplement: Supplement 1 [file NIHPP2026.03.12.711328v1-supplement-1.pdf]

bioRxiv preprint doi: https://doi.org/10.64898/2026.03.12.711328; this version posted March 16, 2026. The copyright holder for this preprint (which was not certified by peer review) is the author/funder, who has granted bioRxiv a license to display the preprint in perpetuity. It is made available under aCC-BY 4.0 International license.

5 Supplementary Notes

Supplementary Note 1: λ_{BMI} selection

The BMI penalty weight λ_{BMI} controls the trade-off between reconstruction fidelity and coexpression-based regularization in the scAttnVI loss (Equation 4). At $\lambda_{\mathrm{BMI}} = 0$ the model reduces to standard scVI; as λ_{BMI} increases, the latent space is increasingly shaped by BMI-derived neighborhood structure rather than reconstruction alone. We therefore choose λ_{BMI} by balancing data fit against regularization.

We select λ_{BMI} for each dataset using *L-curve analysis*[56–57]. The idea is simple: plot the data-fit term (here, reconstruction loss) against the penalty term (normalized BMI penalty) as λ varies. On a log–log scale this curve is typically L-shaped: for small λ, the penalty term decreases rapidly with little reconstruction cost (the vertical arm), whereas for large λ the reconstruction loss grows steeply while the penalty term barely improves (the horizontal arm). The corner, or *elbow*, marks the regime where additional regularization begins to cost more in reconstruction fidelity than it returns in penalty reduction.

bioRxiv preprint doi: https://doi.org/10.64898/2026.03.12.711328; this version posted March 16, 2026. The copyright holder for this preprint (which was not certified by peer review) is the author/funder, who has granted bioRxiv a license to display the preprint in perpetuity. It is made available under aCC-BY 4.0 International license.

We trained scAttnVI on the PBMC dataset (11,990 cells) across 16 values of λ_{BMI}: 0 (scVI baseline), 100, 250, 500, 750, 1,000, 1,500, 2,000, 3,000, 4,000, 6,000, 8,000, 12,000, 16,000, 32,000, and 64,000. Each configuration was trained with three independent random seeds (300 epochs, batch size 512, $n_{\text{latent}} = 10$, $n_{\text{hidden}} = 128$, 1 layer). For each model we recorded the reconstruction loss (ELBO reconstruction component) and the normalized BMI penalty, and evaluated two downstream metrics at full CD8 retention (1,448 cells) and 5% thinning (72 cells): CD8 kNN retention ($k = 30$, fraction of nearest neighbors that are true CD8 cells) and CD8 cluster purity (best Leiden cluster, $k = 12$ neighbors, resolution 1.0).

The L-curve (Extended Data Fig. 1a) exhibits the characteristic L-shape described above[56]. At $\lambda_{\text{BMI}} = 4{,}000$ the reconstruction loss increases by 4.5% relative to scVI ($\lambda = 0$), while the BMI penalty has dropped to 0.0035—well into the diminishing-returns regime where further increases in λ yield little additional penalty reduction. The dual-axis elbow plot (Extended Data Fig. 1b) confirms that $\lambda = 4{,}000$ sits within the elbow range ($\lambda \in [2{,}000, 8{,}000]$, shaded green), where the BMI prior is effective without substantially degrading reconstruction quality. This is the expected L-curve behavior: values below the elbow leave the BMI term underweighted, whereas values above it trade reconstruction fidelity for only small additional regularization gains[57].

At full CD8 retention, $\lambda = 4{,}000$ improves CD8 kNN retention by +0.045 relative to scVI (from 0.870 to 0.916; Extended Data Fig. 1c). Under extreme thinning (5% retention, 72 CD8 cells), the improvement is +0.135 (from 0.291 to 0.426), confirming that BMI regularization specifically benefits rare populations. CD8 cluster purity follows a similar pattern: at 5% thinning, $\lambda = 4{,}000$ improves purity by +0.172 relative to the scVI baseline (Extended Data Fig. 1d).

Both metrics remain stable across the elbow range ($\lambda \in [2{,}000, 8{,}000]$): at full retention, kNN retention varies by < 0.013 and cluster purity by < 0.06 across these four λ values. Under thinning, kNN retention varies by < 0.10 and purity by < 0.08. The biological conclusions of the PBMC analysis (§2.2) are therefore not sensitive to the specific choice of λ_{BMI} within this range.

We recommend selecting λ_{BMI} at the L-curve elbow for each new dataset, using the reconstruction loss and BMI penalty as the two axes. The elbow identifies the regime where the prior is strong enough to anchor rare-population geometry without collapsing the latent space into a BMI-dominated representation. For the PBMC benchmark, values in the range $[2{,}000, 8{,}000]$ all produce qualitatively similar embeddings and downstream results. We chose $\lambda_{\text{BMI}} = 4{,}000$ as the representative value for the analyses in §2.2. Gateway scoring is then performed on latent-space neighborhoods after training, so the chosen λ_{BMI} affects the learned geometry on which the score is evaluated.

Supplementary Note 2: Gateway score diffusion

The raw gateway score can contain isolated high-scoring cells generated by dropout noise or stochastic gene detection. To suppress such outliers, we optionally apply personalized PageRank (PPR) diffusion[14–15] on a k-nearest-neighbor graph ($k = 30$) in the scAttnVI latent space (Equation 7; $\alpha = 0.5$, $T = 3$ iterations). At each iteration, a cell's score is replaced by a convex combination of its own raw score (weight $1 - \alpha$) and the weighted average of its neighbors' scores (weight α). Isolated spikes are therefore attenuated, whereas spatially coherent boundary signal is reinforced. Diffusion is not essential for the analyses reported here; the main-text conclusions remain the same with raw scores.

Extended Data Fig. 2 compares raw and diffused results. Diffusion changes the bell-gene count, but a shared cross-condition bell core remains preserved. WOT concordance is also retained: the odds ratio for enrichment of gateway cells in the top decile of WOT $P(\text{iPSC})$ remains high in both conditions. In UMAP space, most gateway cells are shared between the raw and diffused sets, and cells unique to one set lie in the immediate neighborhood of the shared cells along the MET–iPSC boundary (Extended Data Fig. 2d,e). Across the tested sweep, serum remains nearly flat whereas 2i/LIF becomes more permissive at stronger diffusion; the chosen setting ($\alpha = 0.5$, $T = 3$) lies on the lower-α plateau before that high-diffusion expansion (Extended Data Fig. 2a,b).

bioRxiv preprint doi: https://doi.org/10.64898/2026.03.12.711328; this version posted March 16, 2026. The copyright holder for this preprint (which was not certified by peer review) is the author/funder, who has granted bioRxiv a license to display the preprint in perpetuity. It is made available under aCC-BY 4.0 International license.

Supplementary Note 3: Gateway score threshold selection

The gateway score S_{gateway} is a continuous quantity: every cell receives a value reflecting how strongly it sits at the interface between the iPSC lineage and the dominant non-iPSC fate. Converting this continuous score into a discrete set of gateway cells requires choosing a threshold. Higher thresholds retain fewer cells but sharpen the contrast between the gateway population and its flanking states; lower thresholds include more cells but dilute the boundary signal. We screened a range of thresholds to find the operating point that best balances statistical power with biological specificity, and to confirm that the core findings are robust across this range (Extended Data Fig. 8).

The raw gateway score is extremely sparse: only 0.6% (serum) and 2.2% (2i) of cells receive a non-zero value. After PageRank diffusion with $\alpha = 0.5$ and depth = 3, the score becomes much denser, making the upper tail of the score distribution the relevant operating regime for defining gateway cells. The local rebuild therefore evaluated WOT enrichment and gateway-cell count across 10 thresholds (25th, 50th, 75th, 90th, 95th, 99th, 99.5th, 99.9th, 99.95th, and 99.99th percentiles of S_{gateway}), and recomputed bell-gene summaries for the high-stringency subset (90th, 95th, 99th, 99.5th, 99.9th, 99.95th, and 99.99th percentiles). At each threshold, cells scoring above the cutoff were designated gateway cells.

For each threshold, we recomputed differential expression between the resulting gateway population and both the upstream (MET) and iPSC reference populations (Mann–Whitney U test, Benjamini–Hochberg FDR < 0.05, $|\text{log1p LFC}| > 0.25$). Genes significantly upregulated in gateway cells relative to *both* populations were classified as **bell genes** (peaked expression at the transition); genes downregulated relative to both were classified as **valley genes**.

The relationship between threshold stringency and bell-gene detection reflects a trade-off between biological specificity and statistical power (Extended Data Fig. 8c,d). Bell-gene yield varies with the cutoff, especially in 2i/LIF, but median per-gene effect sizes remain broadly stable across the 99th–99.95th percentile range. At the 99.95th percentile, the local rebuild recovers 83 serum cells and 85 2i/LIF cells, retaining enough power for downstream statistics while still focusing tightly on the boundary-enriched population.

Independent validation by the WOT enrichment odds ratio—measuring enrichment of gateway cells in the top decile of WOT backward-transport $P(\text{iPSC})$—shows the same pattern. OR is near baseline below the 99th percentile, then rises sharply at high stringency (Extended Data Fig. 8a,b). At the 99.95th-percentile operating point, the OR is 29.2 in serum and 251.2 in 2i/LIF. Tightening further to the 99.99th percentile leaves only 17 cells per condition; serum OR increases further and 2i/LIF remains strongly enriched, but the very small sample limits robustness.

Beyond the 99.95th percentile, the number of cells drops to 17 per condition. Although the resulting cells remain strongly enriched for WOT iPSC fate, the small sample limits the stability of downstream gene-level calls. The 99.95th percentile therefore provides the most practical balance between strong orthogonal enrichment and adequate sample size.

Although the total number of bell genes varies with the threshold, the cross-condition overlap remains substantial throughout the high-stringency regime (Extended Data Fig. 8e). At the selected operating point (99.95th percentile), the serum and 2i/LIF analyses share 15 bell genes, and the overlap is larger at looser high-stringency cutoffs. Within this shared set, the epithelial gatekeeper *Sfn* remains present throughout, while tight-junction genes *Cldn6* and *Cldn7* persist through the selected operating point. *Mt1* and *Tuba1b* also remain part of the shared program at the 99.95th percentile, reinforcing the conserved epithelial/stress gateway signature.

The results above show that the 99.95th percentile provides a practical balance between specificity and sample size, and that the gateway framework is robust in the sense that the same core program persists throughout the 99th–99.99th percentile range even though the total bell-gene count changes with threshold. The choice can therefore be adapted to the analytical goal:

bioRxiv preprint doi: https://doi.org/10.64898/2026.03.12.711328; this version posted March 16, 2026. The copyright holder for this preprint (which was not certified by peer review) is the author/funder, who has granted bioRxiv a license to display the preprint in perpetuity. It is made available under aCC-BY 4.0 International license.

Percentile	Cells	Bell genes	Use case
99th–99.5th	1,687–830	66–86	Exploratory; larger cell set for subclustering or trajectory analysis
99.9th	166–169	44–149	Moderate stringency; retains statistical power for most DE tests
**99.95th**	**83–85**	**60–199**	**Default; best balance of specificity and signal strength**
99.99th	17	67–234	Ultra-stringent; very large per-gene effect sizes but small sample

Importantly, the identity of the top-ranked gateway cells is consistent across thresholds: cells selected at the 99.99th percentile are a strict subset of those at the 99.95th, which are in turn a subset of those at the 99.9th. This nested structure means that tightening the threshold does not select a different population, but progressively focuses on the highest-scoring cells within the same boundary region of the latent space.

Supplementary Note 4: Statistical power asymmetry between bell and valley gene detection

Bell genes (transiently upregulated at the gateway) and valley genes (transiently downregulated) are identified by symmetric statistical criteria: both require $|\text{LFC}| > \tau$ and $\text{FDR} < \alpha$ in both comparisons (gateway vs. source and gateway vs. target). Despite this symmetry in the test, the two classes face fundamentally asymmetric detection regimes. Here we derive this asymmetry from first principles, validate it empirically against the WOT reprogramming data, and conclude that bell genes provide the more statistically complete characterization of gateway biology.

Mathematical framework

Setup. Let X_i denote the $\log_2$(1+count)-transformed expression of gene g in cell i:

$$X_i = \log_2(1 + c_i) \geq 0 \tag{8}$$

where $c_i \geq 0$ is the raw UMI count. Expression is **bounded below** by zero and **unbounded above**.

Denote the population means in the three groups as μ_G (gateway, n_G cells), μ_S (source, n_S cells), and μ_T (target, n_T cells). The two log-fold-changes are

$$\text{LFC}_S = \mu_G - \mu_S, \qquad \text{LFC}_T = \mu_G - \mu_T. \tag{9}$$

A gene is classified as *bell* if $\text{LFC}_S > \tau$, $\text{LFC}_T > \tau$, and both pass $\text{FDR} < \alpha$. A gene is classified as *valley* if $\text{LFC}_S < -\tau$, $\text{LFC}_T < -\tau$, and both pass $\text{FDR} < \alpha$. The *detection bottleneck* is

$$A_{\min} = \min\big(|\text{LFC}_S|,\ |\text{LFC}_T|\big). \tag{10}$$

A gene is detected if and only if $A_{\min} > \tau$ and both p-values survive FDR correction. (The reported amplitude uses the mean, $A = (|\text{LFC}_S| + |\text{LFC}_T|)/2$; the analysis below concerns the detection criterion, which depends on the minimum.)

Bounded downregulation, unbounded upregulation.

bioRxiv preprint doi: https://doi.org/10.64898/2026.03.12.711328; this version posted March 16, 2026. The copyright holder for this preprint (which was not certified by peer review) is the author/funder, who has granted bioRxiv a license to display the preprint in perpetuity. It is made available under aCC-BY 4.0 International license.

- **Bell genes.** For a bell gene, $\mu_G > \mu_S$ and $\mu_G > \mu_T$. Since μ_G can be arbitrarily large (no upper bound on expression), the amplitude $A_{\text{min,bell}} = \min(\mu_G - \mu_S,\ \mu_G - \mu_T)$ is bounded only by the maximum achievable expression level: $A_{\text{min,bell}} \in (0, \infty)$.

- **Valley genes.** For a valley gene, $\mu_G < \mu_S$ and $\mu_G < \mu_T$. Since $\mu_G \geq 0$ (Eq. 8),

$$A_{\text{min,valley}} = \min(\mu_S - \mu_G,\ \mu_T - \mu_G) \leq \min(\mu_S,\ \mu_T).$$

  The valley detection bottleneck is **bounded above** by the expression level of the lower-expressing flanking population.

This is the core asymmetry:

$$\boxed{A_{\text{min,bell}} \in (0, \infty), \qquad A_{\text{min,valley}} \leq \min(\mu_S, \mu_T).} \tag{11}$$

Consequence for the dual-comparison bottleneck. For a gene to be detected, *both* $|\text{LFC}_S| > \tau$ and $|\text{LFC}_T| > \tau$ must hold simultaneously. Define the *symmetry ratio*

$$\rho = \frac{\min(|\text{LFC}_S|, |\text{LFC}_T|)}{\max(|\text{LFC}_S|, |\text{LFC}_T|)} \in (0, 1]. \tag{12}$$

Proposition. For a valley gene with fixed total effect $E = |\text{LFC}_S| + |\text{LFC}_T|$, the detection bottleneck is $A_{\text{min}} = \rho \cdot E/(1+\rho)$. The gene passes the threshold τ if and only if $\rho > \tau/(E - \tau)$. As E decreases (due to the expression floor), the minimum required ρ increases—requiring more symmetric contrasts for detection.

Proof. Write $|\text{LFC}_S| = A_{\text{min}}$ and $|\text{LFC}_T| = E - A_{\text{min}}$ with $A_{\text{min}} \leq E - A_{\text{min}}$ (i.e., A_{min} is the bottleneck). Then $\rho = A_{\text{min}}/(E - A_{\text{min}})$, so $A_{\text{min}} = \rho E/(1+\rho)$. The detection criterion $A_{\text{min}} > \tau$ becomes $\rho > \tau/(E - \tau)$. Since valley genes have $E \leq \mu_S + \mu_T$ (bounded), and E is typically smaller for valley than bell candidates (empirically confirmed below), valley genes require higher ρ to pass—but empirically achieve lower ρ. □

Zero-inflation amplifies the asymmetry. Single-cell expression data follows a zero-inflated distribution. Model the expression of gene g in population k as a mixture: $X = 0$ with probability π_k; $X = Y_k > 0$ with probability $1 - \pi_k$. For a valley gene in gateway cells, μ_G is small, implying high dropout ($\pi_G \to 1$). This has two consequences:

1. **Floor compression.** The maximum achievable |LFC| against a reference population with mean μ_{ref} is $\mu_{\text{ref}} - 0 = \mu_{\text{ref}}$ (when $\mu_G = 0$). This ceiling on valley LFC does not exist for bell genes.

2. **Rank-test power loss.** The Mann–Whitney U test operates on ranks. When π_G is high, many gateway cells share the value $X = 0$, creating ties. The asymptotic variance of the U statistic under tied ranks is

$$\sigma_U^2 = \frac{n_G n_{\text{ref}}}{12}\left(n_G + n_{\text{ref}} + 1 - \sum_j \frac{t_j(t_j^2 - 1)}{(n_G + n_{\text{ref}})(n_G + n_{\text{ref}} - 1)}\right)$$

   where t_j is the number of observations tied at rank j. Large tie groups from zero-inflated gateway cells reduce σ_U^2 and thus the test's ability to reject the null.

Empirical validation from WOT data

We test these predictions in the WOT MEF-to-iPSC reprogramming dataset, which contains 83 serum gateway cells and 85 2i/LIF gateway cells drawn from ~165,000 total cells per condition, with ~9,000 genes tested per condition.

bioRxiv preprint doi: https://doi.org/10.64898/2026.03.12.711328; this version posted March 16, 2026. The copyright holder for this preprint (which was not certified by peer review) is the author/funder, who has granted bioRxiv a license to display the preprint in perpetuity. It is made available under aCC-BY 4.0 International license.

Candidate pool asymmetry. Among genes significant in both comparisons (FDR < 0.05), the number of upregulated candidates (both LFCs > 0) far exceeds downregulated candidates (both LFCs < 0):

Condition	Bell candidates	Valley candidates	Ratio
Serum	407	117	3.5×
2i/LIF	1,765	102	17.3×

LFC symmetry confirms the bottleneck. The symmetry ratio ρ (Eq. 12) is significantly lower for valley candidates than bell candidates in both conditions:

Condition	Bell ρ (median)	Valley ρ (median)	P (MW)
Serum	0.907	0.722	2.1×10^{-18}
2i/LIF	0.794	0.653	6.4×10^{-7}

Valley genes are strongly downregulated versus one flanking population but only modestly below the other. The weaker contrast is the bottleneck that prevents detection. For bell genes, both contrasts are nearly equal ($\rho \approx 0.9$), meaning the gateway uniquely expresses these genes at levels exceeding both flanking populations.

Near-miss accumulation. Valley genes accumulate just below the threshold. Define the *near-miss ratio* as the number of genes with $A_{\min} \in (0.75\tau, \tau)$ divided by the number with $A_{\min} > \tau$. In serum, this ratio is 1.28 for valley candidates versus 0.68 for bell candidates (1.9×), consistent with valley genes accumulating at the detection boundary.

Valley genes are genuine. Despite the detection disadvantage, the detected valley genes represent robust biological signal by three criteria. First, all 93 valley genes (58 serum, 46 2i; 11 shared) achieve combined significance $P < 10^{-3}$ by Fisher's method, with median combined $P < 10^{-10}$. Second, of the 58 serum valley genes, a majority show concordant downregulation (both LFCs < 0) in the 2i condition. Third, the 11 shared valley genes, including *Cox6b2*, *Rpl10*, and *Uba52*, yield Fisher's exact OR = 57.9 ($P < 10^{-12}$).

Conclusion

The dual-comparison framework creates an inherent statistical asymmetry: bell gene detection benefits from unbounded upregulation, symmetric LFCs ($\rho \approx 0.9$), and a large candidate pool; valley gene detection is constrained by the expression floor (Eq. 11), produces asymmetric LFCs ($\rho \approx 0.65$), and draws from a 3.5–17.3× smaller candidate pool. We retain identical thresholds for both classes for methodological consistency, and report all detected valley genes for completeness. However, we focus biological interpretation primarily on bell genes, which provide a more statistically powered and biologically coherent characterization of the molecular programs active at fate boundaries.

Supplementary Note 5: Soft-threshold gateway gene analysis

The standard gateway gene analysis uses a hard percentile threshold (99.95th percentile of diffused gateway scores) to define a discrete set of gateway cells, then applies Mann–Whitney U tests to identify bell and valley genes. While effective (Supplementary Note 3), this approach raises a basic question: if we avoid binary gateway membership, can we still detect bell and valley genes that distinguish the gateway from its flanking populations? If not, the gateway cells could simply be an artifact of the cutoff. Here we describe a complementary soft-threshold approach. Instead of a binary gateway membership rule, we compute a weighted gateway-side expression profile from a compact soft-core

bioRxiv preprint doi: https://doi.org/10.64898/2026.03.12.711328; this version posted March 16, 2026. The copyright holder for this preprint (which was not certified by peer review) is the author/funder, who has granted bioRxiv a license to display the preprint in perpetuity. It is made available under aCC-BY 4.0 International license.

of gateway-score cells and compare it with the flanking source and target populations. The answer is yes: the two methods recover the same boundary biology and largely overlapping top-ranked genes.

Rather than averaging across the full positive-score zone, we define a restricted *soft-core* from the upper tail of positive diffused gateway-score cells within each condition (here, the top 5%). This yields 228 serum cells from the 4,550-cell positive-score zone and 274 2i/LIF cells from the 5,479-cell positive-score zone. Each cell i in the soft-core receives a weight proportional to its diffused gateway score: $w_i = s_i / \sum_j s_j$, where s_i is the diffused gateway score. In practice, the gateway-side expression mean becomes a weighted average: higher-scoring soft-core cells contribute more to each gene's gateway mean, while lower-scoring soft-core cells still contribute but with less influence. This retains continuity within a compact transition band while avoiding the much broader positive-score halo used in the separate scVI comparison of Supplementary Note 6.

For each gene, we compute weighted log-fold-changes between the weighted soft-core mean and the unweighted reference populations (MET source and iPSC target cells, randomly subsampled to $n \leq 5{,}000$). Significance is assessed by a weighted Welch t-test with Bessel-corrected weighted variance and Welch–Satterthwaite degrees of freedom. The effective soft-core sample size is 117.1 in serum and 213.9 in 2i/LIF. Bell and valley genes are classified using the same criteria as the standard analysis ($|\text{LFC}| > 0.25$, FDR < 0.05 in both comparisons).

The restricted soft-core analysis identifies 55 bell and 61 valley genes in serum, compared with 60 bell and 58 valley genes from the hard-threshold analysis. In 2i/LIF it identifies 133 bell and 22 valley genes, compared with 199 bell and 46 valley genes from the hard-threshold analysis.

In serum, 40 bell genes are shared between hard and soft-core analyses (Jaccard index = 0.53). Concordance is stronger at the top of the rank list: 60% overlap in both the top 10 and top 20. The leading serum shared bell genes remain *Krt8*, *H19*, *Mt1*, *Car2*, and *Sfn*. For these shared serum bell genes, amplitude is correlated between methods (Spearman $\rho = 0.61$; Extended Data Fig. 9g,h).

In 2i/LIF, 104 bell genes are shared between hard and soft-core analyses (Jaccard index = 0.46), with 50% top-10 overlap and 55% top-20 overlap. Amplitude correlation for shared 2i/LIF bell genes is also retained (Spearman $\rho = 0.51$; Extended Data Fig. 9g,h).

Valley-gene concordance is weaker than bell-gene concordance under the restricted soft-core: 39 serum valley genes and 19 2i/LIF valley genes are shared between hard and soft-core analyses (Extended Data Fig. 9i).

The soft-only and hard-only genes tend to have amplitudes closer to the detection threshold, consistent with borderline genes that cross or miss the significance cutoff depending on the exact restricted weighting scheme. Overall, this analysis supports the same boundary biology while showing that the broad positive-score halo should not be conflated with the compact soft-core reported in Extended Data Fig. 9.

The soft-threshold analysis confirms that the core gateway gene program is robust to the choice of thresholding strategy. The top-ranked bell and valley genes are recovered regardless of whether a hard percentile cutoff or compact soft-core weighting is used, and effect-size estimates are correlated (Extended Data Fig. 9). This concordance supports the detection of gateway populations with distinct programs rather than populations created by one exact cutoff. We nevertheless retain the hard-threshold definition in the main text because it provides a discrete operating set of gateway cells for gene calling, figures, and cross-dataset comparisons.

Supplementary Note 6: WOT scVI baseline comparison

To compare scVI and scAttnVI fairly on the WOT benchmark, we retrained plain scVI on the same filtered serum and 2i/LIF datasets used in the main analysis, using the same gene matrix, latent dimension, and optimization settings but setting $\lambda_{\text{BMI}} = 0$. Gateway scores were then computed with the same downstream pipeline used for scAttnVI: identical source and target annotations, the same kNN-based pull score, the same PPR diffusion parameters, and the

bioRxiv preprint doi: https://doi.org/10.64898/2026.03.12.711328; this version posted March 16, 2026. The copyright holder for this preprint (which was not certified by peer review) is the author/funder, who has granted bioRxiv a license to display the preprint in perpetuity. It is made available under aCC-BY 4.0 International license.

same hard-threshold percentile within each condition. Because the two models learn different latent spaces, UMAPs were generated separately for visualization; the comparison in Extended Data Fig. 11 therefore asks whether each model isolates a compact transition zone and recovers the associated bell-gene signal, not whether the two embeddings are geometrically identical.

Plain scVI still revealed a MET–iPSC transition corridor in both serum and 2i/LIF (Extended Data Fig. 11a–d). The broad transition is therefore not created de novo by BMI regularization. The difference emerges in how sharply that transition is resolved. Under soft scoring, the positive-score zone expanded to 93,489 cells in serum and 158,608 cells in 2i/LIF for scVI, compared with 4,550 and 5,479 cells for scAttnVI. scVI thus captured the overall reprogramming corridor, but it spread that signal across a much broader population.

At the hard threshold, both models return the same number of gateway cells by construction because the percentile cutoff is matched within each condition. Bell-gene recovery is therefore the informative comparison. In serum, scVI recovered 8 hard bell genes, whereas scAttnVI recovered 60; in 2i/LIF, it recovered 57 versus 199 (Extended Data Fig. 11e). For the soft comparison in Extended Data Fig. 11f, we used the full positive-score transition zone ($s_i > 0$ after diffusion) with gateway-score weights, rather than the restricted soft-core analysis used in Extended Data Fig. 9. Under this full-zone weighted analysis, scVI recovered 2 versus 51 bell genes in serum and 1 versus 65 in 2i/LIF. These results indicate that reconstruction alone is sufficient to reveal the broad transition, whereas BMI regularization improves the local resolution needed to separate a compact gateway population and recover the transient genes that peak there.

Supplementary Note 7: Comparison with CellRank and Palantir in WOT

CellRank and Palantir provide an important point of reference because they recover the same broad reprogramming landscape that gateway analysis interrogates. Palantir places cells on a diffusion manifold, orders them from an early root, and estimates branch probabilities and entropy with respect to terminal outcomes. CellRank builds a directed Markov chain on the cell graph and computes absorption probabilities to terminal states. These probabilities can likewise be summarized as entropy. In both methods, high entropy marks cells whose eventual fate remains unresolved.

For WOT, the biologically appropriate terminal comparison is stromal versus iPSC, because this is the dominant successful-versus-failed split described in the original reprogramming study. MET is therefore treated as an intermediate state on the route to reprogramming rather than as a terminal fate. This makes the comparison informative but not identical. A high-entropy CellRank or Palantir cell is not, by construction, a MET–iPSC gateway cell. It is a cell with uncertain terminal outcome. Gateway analysis asks a more localized question: which rare cells lie specifically at the interface between two annotated flanking states, here MET and iPSC, and which genes peak only in that interval.

Extended Data Fig. 12 shows both the common ground and the added resolution. In serum (Extended Data Fig. 12a–c), Palantir partially recovers the same MET–iPSC corridor as the hard gateway, with a Jaccard overlap of 0.169 and a median backward WOT $P(\text{iPSC})$ of 1.03×10^{-5}, indicating partial but incomplete recovery of the fate-enriched corridor. Palantir also recovers a related transient program, including *Krt8*, *Mt1*, *H19*, *Car2*, *Lgals1*, and *Tpm1*, and shares 9 of the top 20 bell genes with the gateway result. CellRank is weaker in serum, with a Jaccard overlap of 0.012, a lower median $P(\text{iPSC})$ of 7.79×10^{-6}, only 12 bell genes, and 1 of the top 20 bell genes shared with gateway analysis. These serum results support the reality of the transition corridor and show that gateway cells lie within a bona fide region of elevated fate uncertainty.

The distinction becomes clearer in 2i/LIF (Extended Data Fig. 12d–j). Here, neither CellRank nor Palantir recovers the same localized hard gateway cell set, and both have zero cell-level overlap with the gateway under the matched-size selection used in this analysis. Palantir remains weakly fate-enriched, with a median backward WOT $P(\text{iPSC})$ of 4.99×10^{-6} and only 12 bell genes, while CellRank broadens into a distinct high-entropy field with a lower

bioRxiv preprint doi: https://doi.org/10.64898/2026.03.12.711328; this version posted March 16, 2026. The copyright holder for this preprint (which was not certified by peer review) is the author/funder, who has granted bioRxiv a license to display the preprint in perpetuity. It is made available under aCC-BY 4.0 International license.

median $P(\text{iPSC})$ of 3.57×10^{-6}. Most importantly, the recovered gene programs diverge. The 2i/LIF gateway retains a large bell-gene set (199 genes) headed by *Mt2*, *Mt1*, *Mylpf*, *Tdh*, *Dppa3*, and *Tuba1b*, whereas CellRank is dominated by broader stromal- and matrix-associated genes such as *Col1a1*, *Col1a2*, *Bgn*, *Sparc*, and *Timp3*, and Palantir yields only a small, distinct bell set led by *Tuba1a*, *Rangrf*, and *Atp5k*.

This added resolution is also reflected in cross-condition coherence (Extended Data Fig. 1 2j). The serum and 2i/LIF gateway analyses share 15 bell genes overall and 4 of their top 20 bell genes (*Mt1*, *Sfn*, *Dstn*, and *Tuba1b*). By comparison, Palantir shares 1 bell gene overall and 1 of its top 20 bell genes across conditions, and CellRank shares 0 bell genes overall and 0 of its top 20 bell genes. Gateway analysis is therefore not only more localized within each condition, but also more coherent across reprogramming contexts. Together, these comparisons show that gateway cells are not an isolated artifact of one model. They sit within the same transition corridor identified by global fate-mapping methods. Gateway analysis, however, resolves that corridor into a rarer and more coherent local interface with a sharper transient gene program.
